# The RNA Silencing Pathway: The Bits and Pieces That Matter

**DOI:** 10.1371/journal.pcbi.0010021

**Published:** 2005-07-29

**Authors:** Marian A. C Groenenboom, Athanasius F. M Marée, Paulien Hogeweg

**Affiliations:** Theoretical Biology and Bioinformatics, University of Utrecht, Utrecht, The Netherlands; Princeton University, United States of America

## Abstract

Cellular pathways are generally proposed on the basis of available experimental knowledge. The proposed pathways, however, may be inadequate to describe the phenomena they are supposed to explain. For instance, by means of concise mathematical models we are able to reveal shortcomings in the current description of the pathway of RNA silencing. The silencing pathway operates by cleaving siRNAs from dsRNA. siRNAs can associate with RISC, leading to the degradation of the target mRNA. We propose and analyze a few small extensions to the pathway: a siRNA degrading RNase, primed amplification of aberrant RNA pieces, and cooperation between aberrant RNA to trigger amplification. These extensions allow for a consistent explanation for various types of silencing phenomena, such as virus induced silencing, transgene and transposon induced silencing, and avoidance of self-reactivity, as well as for differences found between species groups.

## Synopsis

Current descriptions of cellular and molecular pathways, proposed on the basis of available experimental knowledge, are often inadequate in describing the phenomena they are supposed to explain. The authors use mathematical models to reveal shortcomings in the current description of the pathway of RNA silencing. Understanding the mechanism of RNA silencing is of utmost importance, since it is rapidly evolving into a powerful tool in biology and medicine. The authors prove that the generally accepted pathway cannot explain sustained silencing, the prevention of the destruction of a cell's own RNA, and the process of transgene-induced silencing. They propose and analyze specific extensions to the pathway, which do allow for a consistent explanation for these various types of silencing phenomena. These extensions differ in the dynamics they predict, which now can be used to experimentally discriminate between them. The authors' results demonstrate that it is indispensable to check with mathematical models the feasibility of plausible models of cellular and molecular pathways, a step that is usually left out.

## Introduction

RNA silencing protects the eukaryotic cell against viruses and transposons. Viruses produce double-stranded RNA (dsRNA) during reproduction, which can trigger the silencing of viral RNA [1,2]. RNA silencing can also be triggered by a sufficiently high expression of transgenes, a mechanism known as co-suppression or transgene induced silencing [3–6]. The activation of transgene induced RNA silencing is directly linked to the activity of RNA directed RNA polymerase (RDR): overexpression of RDR significantly reduces the number of transgenes needed to induce RNA silencing [7]. RNA silencing deficient mutants show enhanced expression of transposons [8,9]. Transposons could trigger RNA silencing for two possible reasons: they often have multiple inverted repeats (IRs) that form dsRNA transcripts [10], and their high copy number could trigger silencing.

**Figure 1:**
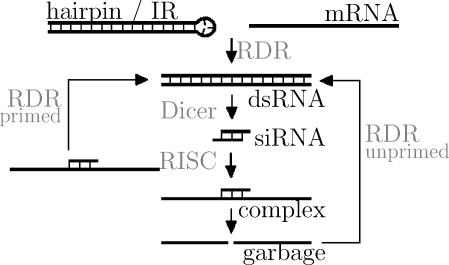
The Standard Pathway of RNA Silencing The figure is based upon Figure 1 in Hutvágner et al. [48].

The currently proposed pathway of RNA silencing is shown in Figure 1. Generally, the process is initiated by the cleavage of dsRNA by Dicer. Dicer, an RNase III-class enzyme, processes dsRNA into small interfering RNAs (siRNAs) 21–25 nucleotides long. siRNAs can then be incorporated into the RNA induced silencing complex (RISC) and “guide” the complex via antisense base-pairing. This results in cleavage of the target mRNA near the center of the siRNA. We refer to the aberrant pieces of RNA after cleavage as “garbage RNA”. Sijen et al. [11] found that a substantial fraction of the siRNAs in *Caenorhabditis elegans* is not derived directly from the introduced dsRNA. To explain this, two amplification routes have been proposed: primed and unprimed amplification [12–14]. In both cases, RDR synthesizes dsRNA: in the case of primed amplification siRNA binds to mRNA to initiate dsRNA synthesis, whereas in the case of unprimed amplification the mere presence of aberrant garbage RNA is sufficient to trigger RDR. In short, the generally accepted pathway of RNA silencing consists of the degradation of mRNA via RISC and an amplification pathway through RDR.

Although it sounds reasonable that such a pathway would suffice to mount responses against both viruses and transposons, we show that the proposed pathway has severe limitations. We will show that it cannot correctly describe observations on transient and sustained silencing and dose dependency. Moreover, such a pathway would be extremely vulnerable for mounting responses against self. Finally, we will show that it cannot describe transgene induced silencing at all. We will then propose three different additions to the mechanism: (i) a siRNA degrading RNase; (ii) primed amplification of garbage RNA; and (iii) activation of RDR dependent on the number of garbage RNAs. The proposed models each give a consistent explanation for various types of silencing phenomena, that is, virus induced silencing, transgene and transposon induced silencing, protection against self-reactivity, as well as for differences found between species groups. The extensions, however, do differ in the dynamics they predict, which could be used to experimentally discriminate between them.

## Results

### Biological Background and Description of Core Model

We study the RNA silencing pathway using concise differential equation models with mass action kinetics. There is strong evidence that there is a common core pathway of RNA silencing present in all organisms capable of RNA silencing. We focus on the experimentally derived common core of RNA silencing (Figure 1 and Introduction), which is the basis for our model. We directly translate this pathway into a system of four coupled ordinary differential equations,


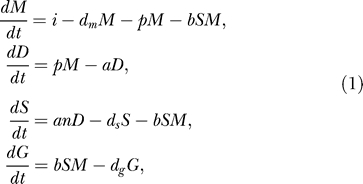


in which *M, D, S,* and *G* describe the number of mRNA, dsRNA, siRNA, and aberrant garbage pieces, respectively. mRNA is transcribed with a rate *i* and degraded with a rate *d_m_*. dsRNA is synthesized from mRNA by RDR with a small rate *p*, and is cleaved into *n* siRNAs with rate *a*. siRNA can associate with mRNA via RISC with rate *b*. For simplicity, we do not implement the formation of RISC explicitly in our model; instead, the siRNA–mRNA complex is directly degraded into aberrant garbage RNA. *d_s_* and *d_g_* describe the degradation of siRNAs and aberrant garbage pieces, respectively.

dsRNA can also enter the pathway in ways other than through RDR: a virus can produce dsRNA, dsRNA can be introduced or injected, or a transcript with IRs can form dsRNA. We simulate the introduction of dsRNA by a stepwise intracellular increase of the amount of dsRNA.

To allow for the formation of secondary siRNAs, we extend the model with the two amplification pathways:


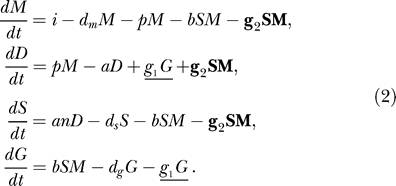


The underlined term *g_1_G* describes the unprimed amplification—the synthesis of dsRNA from aberrant garbage RNA by RDR; and the bold term *g_2_SM* describes primed amplification—the synthesis of dsRNA primed by the presence of a siRNA on mRNA. We consider the pathway with and without the amplification terms.

### Dynamics of Core Model

**Figure 2:**
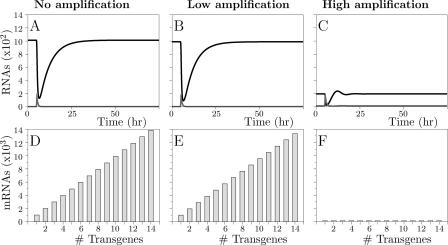
Dynamics of the Standard Models (A) and (B) show that after dsRNA introduction, only transient responses are possible for the standard model without or with low amplification, whereas (C) shows that with high amplification, an arbitrary amount of dsRNA causes sustained silencing. Grey lines indicate dsRNA levels, black lines mRNA levels. (D) and (E) show that an increase in copy number leads to a proportional increase in mRNA levels for the model without or with low amplification, whereas (F) shows that mRNA levels have become independent of copy number in the model with high amplification. RNA levels are expressed in number of molecules per cell. Parameter values can be found in Table 1.

The behavior of the pathway as modeled above is shown in Figure 2. The upper panels show the effect of introducing dsRNA, homologous to an endogenous gene. We first study the model without amplification, which should be representative for mammals, in which RDR has not been found [15]. In mammals, dsRNA or siRNAs have to be continuously supplied to keep a gene silenced. In accordance, the model without amplification allows only for transient responses: siRNAs derived from the dsRNA cause a strong decrease in the amount of mRNA, after which the default equilibrium is re-established (Figure 2A). Since the system has only one attractor, the cell will always return to this attractor, which is the state with normal levels of mRNA. Only when dsRNA is continuously supplied, the gene stays silenced.

**Table 1:**
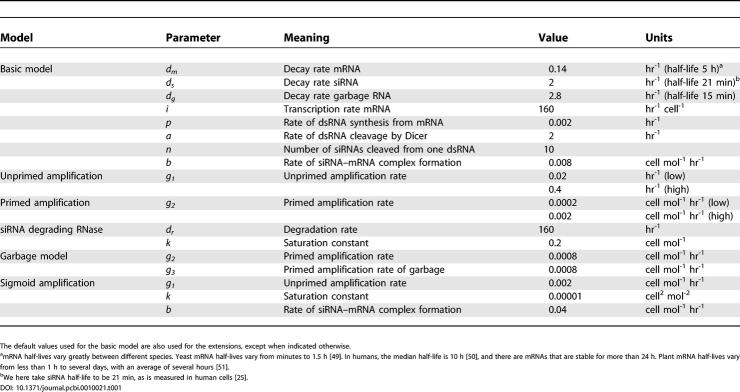
Parameter Values Used in the Models (mol^−1^ Means per Molecule)

The default values used for the basic model are also used for the extensions, except when indicated otherwise.

^a^mRNA half-lives vary greatly between different species. Yeast mRNA half-lives vary from minutes to 1.5 h [49]. In humans, the median half-life is 10 h [50], and there are mRNAs that are stable for more than 24 h. Plant mRNA half-lives vary from less than 1 h to several days, with an average of several hours [51].

^b^We here take siRNA half-life to be 21 min, as is measured in human cells [25].

Amplification of the response via RDR is observed in nematodes, plants, slime molds, and fungi. The dynamics of the core model with primed or unprimed amplification are very similar; we therefore show only the results obtained for primed amplification. At a low amplification rate, the dynamics do not differ from the model without amplification (Figure 2B), but at a high amplification rate the default equilibrium becomes unstable, resulting in perpetual silencing (Figure 2C). Although the cell will remain in the default state as long as siRNAs and dsRNA are completely absent, a single dsRNA strand or siRNA suffices to trigger silencing.

A model of RNA silencing should also be able to explain transgene induced silencing. We therefore analyzed the effect of increasing the number of gene copies. We here assume that each gene copy has the same transcription rate, given by parameter *i*. In the model without or with a low rate of amplification, an increasing copy number leads to a proportional increase in mRNA levels (Figure 2D and E). In contrast, when the amplification rate is high, the amount of mRNA does not depend on the number of gene copies (Figure 2F). In this regime, the cell is always in the silenced state, and therefore the amount of mRNA per cell cannot increase. Thus, transgene induced silencing is not possible in the core model, whether or not amplification is taken into account.

### Deficiencies of Core Model

The core model without amplification is capable of explaining only transient responses. In contrast, in plants RNA silencing can be sustained even after removal of the trigger [16,17], and in *C. elegans* silencing can persist for even more than one generation [18]. Intuitively it seemed plausible that amplification of the response could solve this problem. The core pathway with amplification, however, results in all-or-none type of behavior: either sustained silencing is impossible, or a single dsRNA strand or siRNA is sufficient to trigger perpetual silencing. This actually means that the dynamics of the core pathway with amplification imply inevitable destruction of self.

This problem of self-destruction has also been observed by Bergstrom et al. [19]. In their model study, they added unidirectional amplification, to obtain a transient silencing response. Amplification in plants, however, can be bidirectional [20], so unidirectionality cannot be the sole mechanism that prevents responses to self. Moreover, although unidirectional amplification can prevent sustained responses, it will not prevent transient responses directed against self, implying the unrealistic scenario of an infinite series of auto-destructive responses.

Another major deficiency of the core pathway is that it cannot describe or explain transgene induced silencing. Mathematical analysis of the equations shows that the incapability of transgene induced silencing and the all-or-none type of behavior are inherent properties of the core pathway (see Materials and Methods): the qualitative dynamics do not change when some or all of the mass action terms in the models are replaced by Michaelis-Menten kinetics.

We conclude, that to alleviate the limitations discussed above, the core model should be qualitatively altered. A qualitative difference could be either a missing step in the pathway, or some cooperative effect between RNAs. On the other hand, taking, for example, more details of the RISC complex formation into account, would not make the model qualitatively different, and, therefore, the model would still suffer from the same limitations. That is, this model study shows that the core pathway, which is generally presented as being the basic mechanism, with extensions of the pathway simply being (subtle) modifications of it, is essentially incomplete, and can therefore not be considered to be the core of the pathway.

### Biological Background and Description of Extended Models

We aim to find extensions to the core pathway that are able to provide insight in the type of interactions needed to explain the complexity of RNA silencing. These extended pathways should be able to describe dose dependent responses; the possibility of both transient and sustained responses; transgene or transposon induced silencing; and avoidance of self-reactivity. All extended models need to include at least one of the amplification pathways in order to account for secondary siRNAs and to allow for sustained silencing.

In the first extension, we propose that in addition to the non-specific siRNA degradation a specific siRNA degrading RNase with saturating kinetics is involved (“RNase model”). Such a protein has recently been found in *C. elegans* [21]. We assume that the RNase has Michaelis-Menten kinetics:


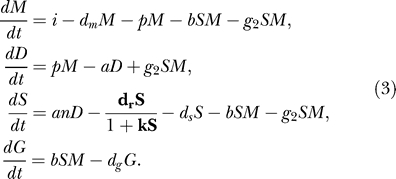


The maximum rate of siRNA degradation by the RNase is given by 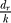. The non-specific degradation of siRNAs has to be included in the RNase model: since the RNase has a saturated response, the siRNA levels would go to infinity without this non-specific degradation.

In our second extension, we generalize the primed amplification process. Whereas in the standard model the process was limited to the amplification of mRNA, we assume here that siRNAs can also bind to garbage mRNA to trigger dsRNA synthesis (“garbage model”):


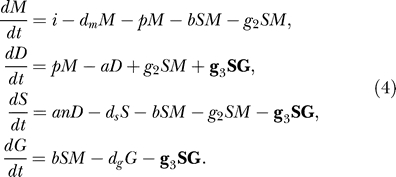


The rate of dsRNA synthesis by primed amplification of garbage RNA is given by *g_3_*.

As a third extension, we consider a revised, unprimed amplification. We explore the possibility that either RDR is activated by the presence of garbage RNA, or that there is another form of cooperation between garbage RNA pieces and RDR. This has been implemented by replacing the mass action unprimed amplification by a sigmoid (unprimed) amplification (“sigmoid model”):


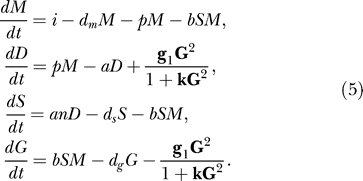


The maximum rate of unprimed dsRNA synthesis by RDR is given by 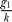.

### Dynamics of Extended Models

The problem with the primed and unprimed amplification in the core pathway is that the number of secondary siRNAs per primary siRNA is basically independent of the initial dose. Consequently, amplification either results in explosion of the reaction, in the case that the number of secondary siRNAs per primary siRNA is larger then one, or the reaction will die out, in the case that the number of secondary siRNAs per primary siRNA is smaller than one. In contrast, in the extended pathways the number of secondary siRNAs becomes dose dependent by introducing a positive feedback into the system. In the RNase model, dose dependency is caused by the saturation of the siRNA degrading RNase: small numbers of siRNAs are rapidly degraded by the enzyme, while at larger numbers the enzyme becomes saturated, which leads to larger amounts of secondary siRNAs. In the garbage and sigmoid model, the cooperation between garbage and siRNAs, and between garbage pieces themselves, respectively, lead to dose dependency.

**Figure 3:**
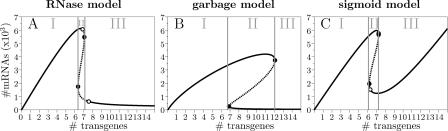
Bifurcation Diagrams of the Extended Models Showing Transgene Induced Silencing Solid lines indicate stable equilibria; dashed lines unstable equilibria; open circles Hopf bifurcations; and closed circles fold bifurcations. The dynamic behaviors in regions I, II, and III are shown in Figure 4.

The behavior of the extended models is more complex than the core model. We can distinguish three main regions of qualitatively different behavior. One way to switch the system to another qualitatively different behavior is by changing the number of gene copies present in the cell. The bifurcation diagrams with the three regions for all three extended models are shown in Figure 3A, B, and C. Plotted is the equilibrium amount of mRNA against the number of copies of a gene; a stable equilibrium is indicated with a solid line, an unstable with a dashed line. In region I, there is only one attractor: after a perturbation, the system will always return to this attractor. In region II, there are two attractors, and the system can end up in either one of them. In region III, there is again only one attractor. We will first discuss each region separately, with the corresponding types of dsRNA induced silencing, and then we will continue discussing the bifurcation diagram as a whole, to understand the process of transgene induced silencing.

**Figure 4:**
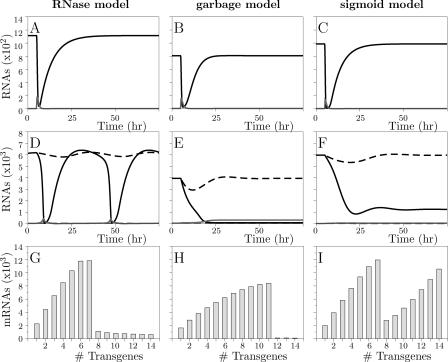
Dynamics of the Proposed Models Grey lines indicate dsRNA levels, black lines mRNA levels. (A), (B), and (C) show transient silencing after dsRNA introduction in the RNase, garbage, and sigmoid model, respectively. (D), (E), and (F) show timeplots of the behavior in the bistable region after introduction of dsRNA: a low dose has only a small effect (dashed lines), but a high dose of dsRNA causes sustained silencing or, in the RNase model, large oscillations (solid lines). (G), (H), and (I) show bar graphs of transgene induced silencing, in the RNase, garbage, and sigmoid model, respectively. Parameter values can be found in Table 1.

In the first region, when there are few copies present, there is only one stable equilibrium. In this default equilibrium, there are low numbers of siRNAs and dsRNA. In this region, mRNA can be silenced transiently by the introduction of homologous dsRNA (Figure 4A, B, and C): when dsRNA is introduced, siRNAs derived from dsRNA cause a strong, rapid decrease of the amount of mRNA, after which the default equilibrium is slowly re-established. Transient silencing after dsRNA injection has been observed in nematodes, flies, and zebrafish [22–24]. Unlike the core pathway, the extended pathways are stable in face of responses against self: a low dose of dsRNA will cause a smaller response than a high dose, and a single dsRNA strand has a negligible effect. This is due to the fact that the amplification in all extended pathways is flux dependent. It means that as long as the copy number is not too high, a low dose of dsRNA will always result in only a small response, and sustained silencing cannot be triggered.

The second region, with an intermediate copy number, is bistable; that is, there are two attractors: the default state and the silenced state. (There is a third equilibrium, which is of the saddle type. The stable manifold of the saddle separates the basins of attraction of the two stable equilibria.) When starting in the default state (dsRNA and siRNAs are almost completely absent), the introduction of a small dose of dsRNA will cause a transient silencing response, after which the default equilibrium is re-established (see Figure 4D, E, and F, dashed lines). A high dose of dsRNA, however, can bring the system from the default equilibrium into the basin of attraction of the silenced equilibrium, which means that sustained silencing is triggered (Figure 4D, E, and F, solid lines). Sustained silencing has been demonstrated in *C. elegans,* where silencing can persist and even be transmitted to the next generation [18]. Also in plants infected with a virus carrying a gene homologous to a plant gene, silencing of the endogenous gene persists even after removal of the virus [16]. The silencing response in plants can also be transmitted via grafting with very high efficiency from silenced stocks to non-silenced stocks [17].

The existence of two attractors prevents undesired sustained responses: only when the amount of dsRNA exceeds a threshold value is the sustained response mounted. Unfortunately, until now few experiments have focused on the correlation between the dsRNA dose and the duration of the silencing response. Lipardi et al. [12] showed that in *Drosophila* embryo extract, doses below a threshold concentration failed to induce RNA silencing, while ten times higher doses were able to trigger silencing. This study indicates the existence of a threshold; the duration of the response, however, has not been investigated. The results of Li et al. [24] also indicate the existence of a threshold: they showed that in zebrafish (*Danio rerio)* embryos small doses of dsRNA lead to partial phenotypic changes only, while high doses of dsRNA lead to more than 50% partial and 35% full phenotypic changes. The partial phenotypic effects could indicate that there was only a transient response in the embryos, while the full phenotypic changes triggered by a high dose of dsRNA could indicate a sustained response.

In the garbage model, the amounts of mRNA, dsRNA, and siRNAs are stable in both attractors, but in the RNase and the sigmoid model, oscillations can occur in this region. The oscillations around the default equilibrium are always of small amplitude, but around the silenced equilibrium they can become large. The region with oscillations is much smaller in the sigmoid model than in the RNase model. We therefore show dynamics with oscillations for the RNase model and without oscillations for the sigmoid model (Figure 4D and F, respectively).

Finally, in the third region, with a high copy number, only the silenced state, with low levels of mRNA and high levels of siRNAs, is stable. The introduction of additional dsRNA will have only a small effect on the already largely reduced amount of mRNA. When a gene is present at very high copy numbers, its mRNA will be silenced continuously.

Technically, the transitions between the regions can be characterized by different bifurcations. In the garbage model, the default state and the silenced state disappear due to fold bifurcations (Figure 3, closed circles) when the number of transgenes is respectively increased or decreased. In the other two models, shortly before the fold bifurcations the equilibria become unstable due to Hopf bifurcations (Figure 3, open circles), which leads to oscillatory behavior around the equilibria. These oscillations then disappear due to homoclinic connection bifurcations. When, by changing the number of transgenes, a fold or homoclinic connection bifurcation is passed, the dynamics immediately jump to the other equilibrium (or around it, in the case that there are oscillations).

The bifurcation diagram depicts the process of transgene induced silencing. In Figure 4G, H, and I we plot bar graphs, where each bar indicates the equilibrium amount of mRNA for a certain number of gene copies, to compare our results to the graphs obtained experimentally in *Drosophila* by Pal-Bhadra et al. [5]. They inserted one to ten copies of a full *Adh* transgene using different insertion sites. Stocks with the same copy number showed similar mRNA levels, independent of where the genes were inserted into the genome. One copy resulted in a normal amount of mRNA, while up to five copies, an extra copy resulted in a proportional increase in mRNA levels. However, when a sixth copy was inserted, RNA silencing was triggered, indicated by the presence of siRNAs, and dramatically decreased mRNA levels. At even higher copy numbers, the amount of mRNA was lying around the amount expected for one or two copies only. The bar graphs we obtained with our models are in close correspondence: the amount of mRNA in the cell initially increases with increasing numbers of transgenes; however, when the number of transgenes is increased beyond a threshold level, RNA silencing is triggered.

### Parameters Dependence and Predictions

There is only a limited amount of experimentally measured parameters available, which are generally obtained for different model organisms. Moreover, the range of measured values can often be very large. We, therefore, do not focus on specific parameter values, but instead use mean values to show the qualitative dynamics. We then vary the parameter values and infer what kind of qualitative and quantitative changes are to be expected to accompany such parameter changes. When data are available, we compare these model predictions with experiments in which specific parameters have been varied.

The default parameters are given in Table 1. We assumed stable mRNA (half-life 5 h), a 20× faster decay of garbage pieces (half-life 15 min), and slightly more stable siRNAs (half-life 21 min, as measured in human cells [25]). The other parameters are chosen such as to depict the full capabilities of the models.

**Figure 5:**
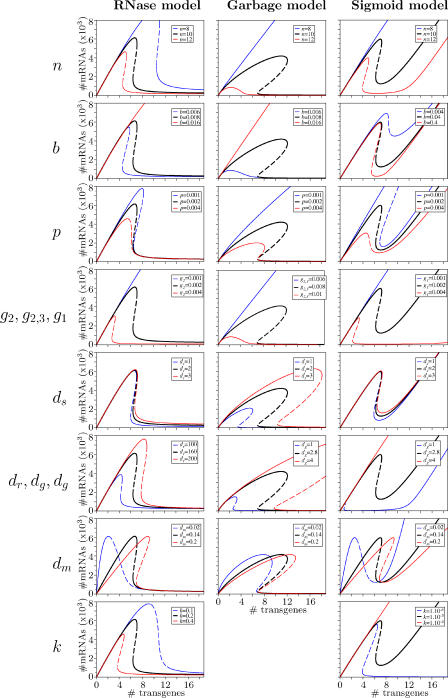
Changes in the Bifurcation Diagrams of Figure 3 Due to Changing Parameter Values The black lines indicate the standard parameter values, the blue lines a lower value, and the red lines a higher value for the corresponding parameter.

We distinguish five types of qualitatively different effects that can be caused by changing parameter values (Figure 5). The changes in behavior can be described in terms of the threshold, which is the number of transgenes needed to trigger silencing; and in terms of the bistable point, which is the lower bound of the bistable region. Note that in the sigmoid model, the amount of mRNA in the cell always increases again at high copy numbers.

Type I behavior occurs in the garbage model when changing parameters *n, b,* and *g,* and in all three models when changing *p*. Changing these parameters does not influence the bistable point (that is, the value above which sustained silencing can be triggered), but moves the threshold to different copy number and mRNA levels. This means that the size of the bistable region changes: when the threshold is lower than the bistable point, there is no bistable region, and transgene silencing is triggered at low copy numbers. When the threshold becomes very high, the bistable region is very large: only a very high copy number triggers silencing, while sustained silencing triggered by the introduction of dsRNA becomes possible in a large region.

Type II behavior is typical for the RNase and sigmoid model. In both models, changes in the parameters *n, b, g_1,2_,* and *k* result in type II behavior, as well as *d_r_* in the RNase model, and *d_g_* in the sigmoid model. Parameter changes move the bistable point and the threshold in the following manner: a low threshold coincides with an even lower bistable point; a high threshold with an even higher bistable point. This means that, in contrast to type I behavior, the bistable region disappears when the threshold is high, and sustained silencing becomes impossible. In the sigmoid model, also the possibility of transgene induced silencing disappears, since there is no noticeable decrease in mRNA levels beyond the threshold. In the RNase model, this is not the case: mRNA levels will always decrease with sufficiently high copy number, although the threshold number of transgenes can sometimes lie outside of the graphs in Figure 5.

Type III behavior occurs for changes in *d_m_* in all three models. In this case, the threshold moves to different copy numbers, but the amount of mRNA at the threshold remains the same. Instead, the initial slope of the mRNA level changes. The bistable point is not affected, and since the threshold moves, the bistable region can increase or decrease in size (and even disappear).

Type IV behavior is typical for changes in *d_s_* and *d_g_* in the garbage model. These parameters scale the complete bifurcation diagram.

Type V behavior occurs in the RNase and the sigmoid model for changes in *d_s_*. In this case, the only thing that changes is the amount of mRNA just after transgene silencing is triggered.

Changes in the threshold value of gene copies have been experimentally observed. Several studies suggest that the amount of transcribed mRNA plays an important role in the ability of transcripts to trigger transgene induced silencing. When a transgene is under control of a 35S promoter with a double enhancer, the gene is transcribed at such a high rate that a single transgene can be sufficient to trigger silencing [26]. Also, in petunia the strength of the promoter is correlated with the frequency and degree of silencing [27], and plants homozygous for a transgene are much more often silenced than hemizygous plants [27–32].

These observations are consistent with our models: when a gene is more highly expressed (in our models described by a higher value of *i,* the transcription rate), fewer copies are needed to trigger silencing. This is because changing *i* effectively rescales the *x*-axis of the bifurcation diagrams.

Our models suggest that the amount of dsRNA per mRNA is a major factor determining the threshold. When more dsRNA per mRNA is produced, the threshold to trigger transgene silencing will be lower. For example, an increase in the parameter *p,* the rate of dsRNA synthesis by RDR, results in all models in a decrease of the threshold. An increase in amplification *(g)* will have a similar effect. Our results are in line with experimental observations: Forrest et al. [7] showed that in strains that overexpressed RDR, the number of transgenes required to induce silencing is decreased. Likewise, transcripts with tandem IRs, which produce much more dsRNA per mRNA, have shown to be very efficient inducers of RNA silencing [10,33]. Finally, transposons often contain long IRs and are present in high copy numbers, both of which we have shown to induce silencing.

These experimental results are consistent with our models, but they do not make it possible to distinguish between them. Instead, we need experiments in which certain specific parameters are varied. For example, the models predict a completely different effect of the overexpression of RISC (all its components have to be overexpressed). In the garbage and RNase model, RISC overexpression leads to an increase and ultimately disappearance of the threshold. In the sigmoid model, we find the complete opposite: the disappearance of the threshold is not caused by RISC overexpression, but by RISC underexpression (Figure 5).

## Discussion

The extended models each provide a unified framework for different RNA silencing phenomena. They provide consistent explanations for (i) dose dependent dsRNA induced silencing; (ii) stability against self-directed responses; (iii) the dependence of transgene induced silencing on RDR; (iv) the effect of IRs; (v) multiple copies; (vi) efficient promoters; and (vii) the ability of transposons to trigger silencing.

Previously it has been proposed that transgene induced silencing is triggered only if the number of transgenes exceeds a threshold level [5,6,29–31]. It has also been observed that the overexpression of RDR reduces the threshold [7]. Our models support the threshold hypothesis and give a mechanistic explanation for it: we propose that the amount of dsRNA per transcript matters.

The extensions also explain differences in RNA silencing phenomena in different species groups. According to our extended models, in organisms that have RDR homolog(s), such as plants, fungi, nematodes, and cellular slime molds, silencing can be induced by transgenes, IRs, transposons, and dsRNA. In contrast, we have shown here that organisms without RDR are unable to trigger transgene induced silencing. Accordingly, experiments have shown that plants with a mutation in RDR are no longer able to bring about transgene induced silencing, while virus (dsRNA) induced silencing is still possible in these strains [34]. The presence of an RDR in *Drosophila* is currently disputed. Some experiments strongly suggest the presence in *Drosophila* of an RDR or a protein that functions as an RDR [5,12,35]. Other experiments, however, argue against the presence of such an enzyme (a BLAST search, for example, does not yield an RDR homolog) [36–38]. Since high transgene numbers (without IRs) are capable of inducing RNA silencing in *Drosophila,* our model suggests that a protein with the same function as RDR must be present. Mammals lack RDR, and in agreement with our models, they are capable of only transient silencing induced by siRNAs [39] (in mammals, dsRNA triggers several non-specific responses [40,41]; sustained silencing in mammals can be accomplished only by continuous expression of siRNAs).

We did not include the effect of siRNAs on DNA chromatin, which is referred to as transcriptional silencing or heterochromatinization. Transcriptional silencing plays a role in transposon silencing [42–45]. Nolan et al. [46], however, recently showed that in the fungus *Neurospora crassa* the LINE1-like transposon, *Tad,* is post-transcriptionally silenced and not significantly methylated, indicating that transposon induced silencing in *N. crassa* can be independent of DNA methylation. Moreover, also in *Drosophila* transgene induced silencing has been shown to be solely post-transcriptional [5]. Although heterochromatinization can play an important role in transposon silencing, our model study indicates that the addition of heterochromatinization alone—that is, the stop in transcription—to the core pathway will not make transgene silencing possible. Heterochromatinization will only decrease mRNA transcription, and does not provide the necessary positive feedback.

Although it has been shown recently that RISC can perform multiple rounds of cleavage [47], we assume only one cleavage per RISC complex. Adding multiple turnover of RISC, however, does not affect the qualitative behavior of the model (unpublished data).

We proposed three different additions to the pathway. We here suggest some ways of testing or rejecting experimentally the predictions made by the different extensions. In the parameter section, we have already discussed the different behavior of the sigmoid model when RISC is overexpressed. Another difference is that only in the sigmoid model, after silencing is triggered, even higher copy numbers will cause an increase in mRNA levels again. Such an increase, however, can also indicate other sigmoid responses in the pathway, for example in Dicer or RISC. Sigmoid kinetics alone are not able to allow for low mRNA levels when copy numbers become very large. It can be argued, however, that a combination of heterochromatinization with a sigmoid response will be able to keep mRNA levels silenced.

Recently, a siRNA degrading RNase has been found in *C. elegans* [21]. The pathway with the siRNA degrading RNase, however, is able to cause transgene silencing only when it degrades siRNAs very rapidly and when it saturates quickly. In fact, even when this is the case, this pathway only limitedly allows for sustained RNA silencing, because the parameter range for sustained silencing is very small. We would like to see experiments that focus on the correlation between the dose of dsRNA and the duration of the silencing response. Such experiments will give insights into the existence of a positive feedback and will show if there is a bistable region. The next step would be to investigate the dependence of both the threshold and the size of the bistable region on gene copy numbers, and how this depends on changes in parameters. These observations can then be compared with the dependencies predicted in the parameter section.

The garbage model could be tested by investigating the possibility of siRNAs to serve as primers for RDR on aberrant garbage pieces. When that is possible, we expect that this primed amplification of garbage is a missing step in the RNA silencing pathway. It represents only a small addition to the currently known pathway, but it has a large impact on the dynamics, making transgene and transposon silencing, as well as dose dependent sustained silencing, possible for a wide range of parameters. Therefore, we conclude that in RNA silencing it is “the bits and pieces” that matter.

## Materials and Methods

### Mathematical proof of limitations of core pathway.

In this section, we prove that the core pathway, with or without amplification, is incapable of transgene induced silencing and sustained silencing.

In transgene induced silencing, the amount of mRNA in the cell initially increases when the number of transgenes is increased, but a further increase in the number of transgenes leads to a sudden drop in the equilibrium amount of mRNA, due to RNA silencing [5]. This implies that two different copy numbers can lead to exactly the same equilibrium amount of mRNA (see Figure 1 or Figure 3 in [5]). We refer to the equilibrium for a low copy number as the default state, and to the equilibrium for a high copy number as the silenced state. Despite the high transcription of mRNA, in the silenced state the amount of mRNA remains low, due to the high levels of dsRNA and siRNAs that are present. Consequently, when it would be possible to keep the amount of mRNA in the cell constant (cf, patch clamp techniques in neuroscience), there should exist mRNA levels for which there are at least two stable equilibria in the system, each with a different amount of dsRNA. Mathematically, this requirement can be studied by considering the variable *M* as a fixed parameter *m*. Then for a certain interval of values of *m,* there should exist at least two stable equilibria, which implies the existence of at least three equilibria, since one equilibrium will be unstable. We will show here that the previously proposed pathway cannot fulfill this requirement and, consequently, is not able to describe and explain transgene induced silencing.

**Figure 6:**
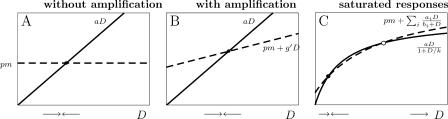
Equilibrium Analysis of the Models Based on the Previously Proposed Pathway Transgene induced silencing requires the existence of two stable equilibria with different dsRNA *(D)* levels for one fixed value of mRNA *(m).* Shown here are the influx (dashed line) and breakdown (solid line) of *D* as a function of *D*. Equilibria are found when influx balances breakdown—that is, where these lines cross. Both without and with amplification, there exists only one stable equilibrium; when mass action terms are replaced by saturated responses, there can be zero, one, or two equilibria, of which at most one is stable. The pathway is therefore unable to describe transgene induced silencing.


The requirement is analyzed by studying the equilibrium dsRNA *(D)* level. Without amplification, the dsRNA dynamics consist of two parts only: a positive influx *(pm)* and a decay by Dicer *(aD).* In Figure 6, we depict the influx and breakdown of *D* as a function of *D*. To obtain an equilibrium, the influx should balance the breakdown—that is, the depicted lines should cross. Hence, without amplification there will be one equilibrium only (Figure 6A). Thus, each gene copy number leads to a unique mRNA level, so it can therefore only be the case that the equilibrium mRNA level monotonically rises with increasing transgene copy number (see Figure 2D). The addition of either primed or unprimed amplification does not allow for an increase in the number of stable equilibria. This can be derived by solving for equilibria by means of putting both *dS/dt* and *dG/dt* to zero:

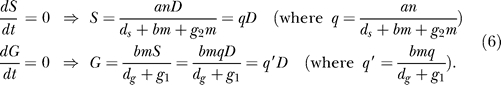


This means that the amplification terms can be written as a linear function of *D,* which can never result in more than one stable equilibrium (Figure 6B):


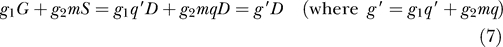


When mass-action terms are replaced by Michaelis-Menten kinetics, the amplification can be rewritten as a sum of saturated responses as well: 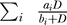. When the breakdown is not saturated, this still trivially leads to one equilibrium only. However, when breakdown by Dicer shows a saturated response, more than one equilibrium can be found (but also no equilibria, in the case that the saturation is very rapid). To allow for at least two stable equilibria, the line 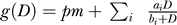 should cross the line 
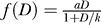
 at least three times, because a second equilibrium will always be unstable. (Note that since in this analysis *m* can be treated as a constant, the term *pm* could as well have a more complicated—for example, saturated—dependency on *m*.) This, however, is never possible. The model can be rescaled to 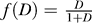. Consider the ratio of both functions:


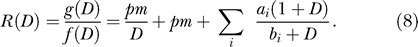


Equilibria are found when *R(D)* = 1; to obtain three equilibria, the derivative of *R(D)* should have at least two roots:


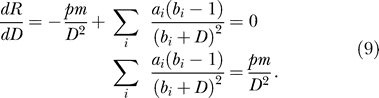


However, by multiplying both sides with the monotonically increasing function (1+*D*)^2^, it can be shown that there is at most one root, since the left-hand side is a monotonically increasing function of *D*:


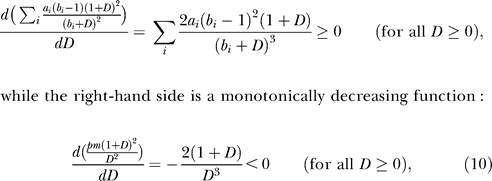


Consequently, *dR/dD* has only one root, and there will be at most two equilibria, from which at most one is stable (Figure 6C). Thus, in the previously proposed pathway transgene induced silencing is impossible.

Likewise, model dynamics describing sustained responses require multiple steady states for a unique set of parameters. This requirement is implicitly equivalent to the previous one, since the existence of a second stable equilibrium (with a lower mRNA level) automatically implies that the same equilibrium mRNA level can be found for a lower transgene copy number. That is, the previously proposed pathway can neither describe nor explain transgene induced silencing, nor sustained silencing triggered by injecting dsRNA.

### Programs used.

The timeplots in Figures 2 and 3 are produced with GRIND, a computer program for the study of differential equation models by means of numerical integration, steady state analysis, and phase space analysis (http://theory.bio.uu.nl/rdb/software.html). The bifurcation diagrams are produced with CONTENT, an integrated environment for bifurcation analysis of dynamical systems (http://www.math.uu.nl/people/kuznet/CONTENT/).

## Abbreviations

dsRNA: double-stranded RNA
IR: inverted repeat
RDR: RNA directed RNA polymerase
RISC: RNA induced silencing complex
siRNA: small interfering RNA
